# Detection of Cetacean Poxvirus in Peruvian Common Bottlenose Dolphins (*Tursiops truncatus*) Using a Pan-Poxvirus PCR

**DOI:** 10.3390/v14091850

**Published:** 2022-08-23

**Authors:** Léa Luciani, Géraldine Piorkowski, Xavier De Lamballerie, Koen Van Waerebeek, Marie-Françoise Van Bressem

**Affiliations:** 1 Unité des Virus Émergents (UVE: Aix-Marseille Univ-IRD 190-Inserm 1207-IHU Méditerranée Infection), 13008 Marseille, France; 2 Cetacean Conservation Medicine Group, Peruvian Centre for Cetacean Research/Centro Peruano de Estudios Cetológicos (CEPEC), Museo de Delfines, Lima 20, Peru

**Keywords:** poxviruses, cetaceans, cetacean poxvirus, Peru, pan-poxvirus PCR

## Abstract

Cetacean poxviruses (CePVs) cause ‘tattoo’ skin lesions in small and large cetaceans worldwide. Although the disease has been known for decades, genomic data for these poxviruses are very limited, with the exception of CePV-*Tursiops aduncus,* which was completely sequenced in 2020. Using a newly developed pan-pox real-time PCR system targeting a conserved nucleotide sequence located within the Monkeypox virus D6R gene, we rapidly detected the CePV genome in typical skin lesions collected from two Peruvian common bottlenose dolphins (*Tursiops truncatus*) by-caught off Peru in 1993. Phylogenetic analyses based on the sequencing of the DNA polymerase and DNA topoisomerase genes showed that the two viruses are very closely related to each other, although the dolphins they infected pertained to different ecotypes. The poxviruses described in this study belong to CePV-1, a heterogeneous clade that infects many species of dolphins (Delphinidae) and porpoises (Phocoenidae). Among this clade, the *T. truncatus* CePVs from Peru were more related to the viruses infecting Delphinidae than to those detected in Phocoenidae. This is the first time that CePVs were identified in free-ranging odontocetes from the Eastern Pacific, surprisingly in 30-year-old samples. These data further suggest a close and long-standing pathogen–host co-evolution, resulting in different lineages of CePVs.

## 1. Introduction

Poxviruses are large, enveloped, double–stranded DNA viruses that replicate in the cytoplasm. The *Poxviridae* family is divided into two subfamilies based on the hosts they infect: the *Entomopoxvirinae* in insects and the *Chordopoxvirinae* in vertebrates. The chordopoxviruses comprise at least 18 genera, including the *Orthopoxviruses* and *Parapoxviruses* [1]. Many have broad host ranges and are zoonotic, such as, for example, *Monkeypox virus* [2]. Several chordopoxviruses infect marine mammals such as pinnipeds, sea otters (*Enhydra lutris*) and cetaceans, mostly inducing cutaneous lesions of variable severity [3]. Though most pinniped poxviruses belong to the genus *Parapoxvirus*, viruses related to the orthopoxviruses have also been detected in a gray seal (*Halichoerus grypus*) [4] and Steller sea lions (*Eumetopias jubatus*) [5]. The sea otter poxvirus belongs to the new genus ‘*Mustelpoxvirus*’ [6], while cetacean pox viruses (CePVs) pertain to a new genus of the *Chordopoxvirinae* that has not yet been recognized by the International Committee on Taxonomy of Viruses [7,8,9].

CePVs cause ‘tattoo skin disease’ (TSD), a dermatopathy characterized by irregular, grey, black or yellowish, stippled skin lesions with an irregular outline that may occur on any part of the body but show a preferential distribution depending on the species [10,11]. The disease has regularly been detected in odontocetes and mysticetes worldwide, with prevalence levels varying from sporadic to high [12]. Recently, the complete sequence of a cetacean poxvirus (CePV-TA) was recovered from tattoo skin lesions sampled in a captive Indo-Pacific bottlenose dolphin (*Tursiops aduncus*) [9]. The virus has one of the smallest poxvirus genomes (121, 769 bp) and a low G + C content (28%). It comprises 120 open reading frames (ORFs), of which 80 encode proteins that are highly conserved among the subfamily *Chordopoxvirinae*. CePV-TA is the only poxvirus that possesses two copies of the gene encoding the E3L protein that, in vitro, inhibit the activation of interferon antiviral enzymes [9]. A phylogenetic analysis showed that CePV-TA proteins form a unique chordopoxvirus branch, basal to the clade comprising members of the genera *Centapoxvirus* and *Orthopoxvirus* and poxviruses of Artiodactyls (*Cervidpoxvirus*, *Suipoxvirus*, *Capripoxvirus*, *Leporipoxvirus* and *Yatapoxvirus*) [9]. A genetic analysis of partial sequences of the CePV DNA polymerase and DNA topoisomerase I genes detected in several cetaceans from the Atlantic Ocean, the North Sea and the Mediterranean Sea indicated that the poxviruses of odontocetes and mysticetes belong to two different clades, namely, CePV-1 and CePV-2, respectively [7,8,9,13,14]. CePV-1 includes strains recovered from Delphinidae (dolphins) and Phocoenidae (porpoises) from North and South America, Europe and Asia [7,8,9,15]. The Delphinidae strains are closely related, independent of the host species and ocean province [8,15]. The Phocoenidae strains, all originating from UK harbor porpoises (*Phocoena phocoena*), form a distinct lineage [8,13].

**Figure 1:**
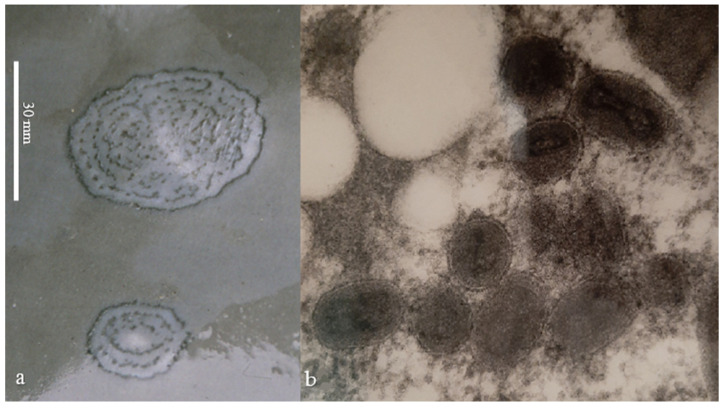
(**a**) Typical tattoo skin lesions in inshore common bottlenose dolphin (*Tursiops truncatus*) MFB-465; (**b**) poxvirus particles in skin lesions of offshore *T. truncatus* (MFB-187) from central Peru (Magnification ≅ × 180.000).

Cetacean poxvirus skin disease is endemic in Peruvian populations of dusky dolphin (*Lagenorhynchus obscurus*), long-beaked common dolphin (*Delphinus* cf. *capensis*), common bottlenose dolphin (*Tursiops truncatus*) (offshore and inshore stocks, *sensu* [16]) and in Burmeister’s porpoise (*Phocoena spinipinnis*) (Figure 1a). Prevalence levels varied between 34.7% in *L. obscurus* and 67.3% in *P. spinipinnis* in 1993–1994. Though viruses with morphological characteristics of poxviruses were repeatedly observed by electron microscopy in diagnostic tattoo skin lesions (Figure 1b) [11,17] and neutralizing antibodies against vaccinia virus were detected in the serum samples of a high percentage of Peruvian dolphins and porpoises [18], attempts to cultivate these poxviruses in vitro in cell cultures and on embryonated eggs failed (M-F. Van Bressem and Malcolm Bennett, unpublished data). Thus, they have not yet been characterized, nor have any cetacean poxviruses from other parts of the Eastern Pacific. In this context, we used a recently developed real-time PCR system for the universal detection of poxviruses (pan-poxvirus) [19] to test for CePV DNA in tattoo skin lesions from dolphins and porpoises caught in fisheries off the coast of Peru in the period of 1989–1995 [20]. This system targets a conserved nucleotide sequence located within the Monkeypox virus D6R gene that belongs to the so-called 49 “core” genes of poxviruses, which are involved in key functions such as replication, transcription, and virion assembly [19,21]. It has a sensitivity of <1000 copies/mL for most genera, and the amplified sequence theoretically allows discrimination at the genus level [19]. This pan-poxvirus PCR system had never been tested on CePVs because the CePV-TA complete genome was not available at the time of its development. Additionally, pan-ortho- and pan-para-poxvirus systems [22,23] that allow the detection of Orthopoxviruses and Parapoxviruses, respectively, were also tested on these samples.

## 2. Materials and Methods

Typical tattoo skin lesions (Figure 1a) were sampled in five odontocete species, including three long-beaked common dolphins, one short-finned pilot whale (*Globicephala macrorhynchus*), five dusky dolphins, one inshore and one offshore common bottlenose dolphin and five Burmeister’s porpoises landed as by-catch at the fish markets of Cerro Azul, Ancón, Pucusana and San José, Peru, between 1989 and 1995 (Table 1). Viruses with the morphological characteristics of poxviruses (Figure 1b) had been demonstrated by electron microscopy in three samples (MFB-001, MFB-187 and KVW-2283; Table 1). Samples of TSD lesions were preserved in 10% formalin, dimethylsulfoxide (DMSO) or 5% glutaraldehyde at room temperature (Table 1).

All samples were washed twice with sterile water, then transferred into 2 mL tubes containing 1 mL of a 0.9% sodium chloride solution and 3 mm tungsten beads and crushed using a TissueLyser II (Qiagen, Hilden, Germany) for 20 min at 30 cycles/s and then centrifuged for 10 min at 16,000× *g*. The supernatant media were transferred into 1.5 mL tubes and centrifuged for 10 min at 16,000× *g*. Then, 60 µL of DNA extract was obtained from 400 µL of doubly centrifuged supernatant using the EZ1 extractor and the corresponding VirusMini kit 2.0 according to the manufacturer’s instructions (Qiagen, Hilden, Germany). Real-time PCR was performed using QuantStudio 12K Flex thermal cyclers (ThermoFischer, Waltham, MA, USA). For the broad-spectrum poxvirus real-time PCR, we used the previously described D6R gene-based system [19] using the QuantiNova SYBR^®^ Green RT-PCR Kit (Qiagen, Hilden, Germany). For real-time PCR targeting the *Orthopoxvirus* or the *Parapoxvirus* genera, we used the E9L gene-based system [22] and the B2L gene-based system [23], respectively, as previously described, using the Invitrogen™ EXPRESS qPCR Supermix universal kit (ThermoFischer, Waltham, MA, USA). To confirm the presence of cetacean poxvirus DNA, we used primers targeting either DNA polymerase or DNA topoisomerase, as previously described [7], using the AmpliTaq Gold DNA Polymerases kit (ThermoFischer, Waltham, MA, USA) and the 2720 thermal cycler (AppliedBiosystem, Waltham, MA, USA). Amplicons were sequenced using next-generation sequencing after Qubit quantification using a Qubit^®^ dsDNA HS Assay Kit and a Qubit 2.0 fluorometer (ThermoFisher Scientific, Waltham, MA, USA). Libraries were built by adding barcodes, for sample identification, and primers to amplicons using the AB Library Builder System (ThermoFisher Scientific, Waltham, MA, USA). To pool the barcoded samples equimolarly, a quantification step by real-time PCR using the Ion Library TaqMan™ Quantitation Kit (Thermo Fisher Scientific, Waltham, MA, USA) was carried out. An emulsion PCR of the pools and loading on a 520 chip was performed using the automated Ion Chef instrument (ThermoFisher, Waltham, MA, USA). Sequencing was performed using the S5 Ion torrent technology (ThermoFisher Scientific, Waltham, MA, USA) following the manufacturer’s instructions. A consensus sequence was obtained after the trimming of reads (reads with quality scores < 0.99 and lengths < 30 pb were removed, and the 25 first and 25 last nucleotides were removed from the reads) and the mapping of the reads on a reference (MF458200 for DNA topoisomerase and MH005249 for DNA polymerase) using CLC genomics workbench software v.21.0.5 (Qiagen, Hilden, Germany). The parameters for reference-based assembly consisted of match score = 1, mismatch cost = 2, length fraction = 0.5, similarity fraction = 0.8, insertion cost = 3 and deletion cost = 3.

The obtained DNA polymerase and DNA topoisomerase sequences and homologous sequences (Table 2) from databases were aligned with ClustalW [24]. MEGA 7 software was used to infer phylogenetic trees using the maximum-likelihood method with a bootstrap analysis (5000 replicates) [25].

## 3. Results

**Figure 2:**
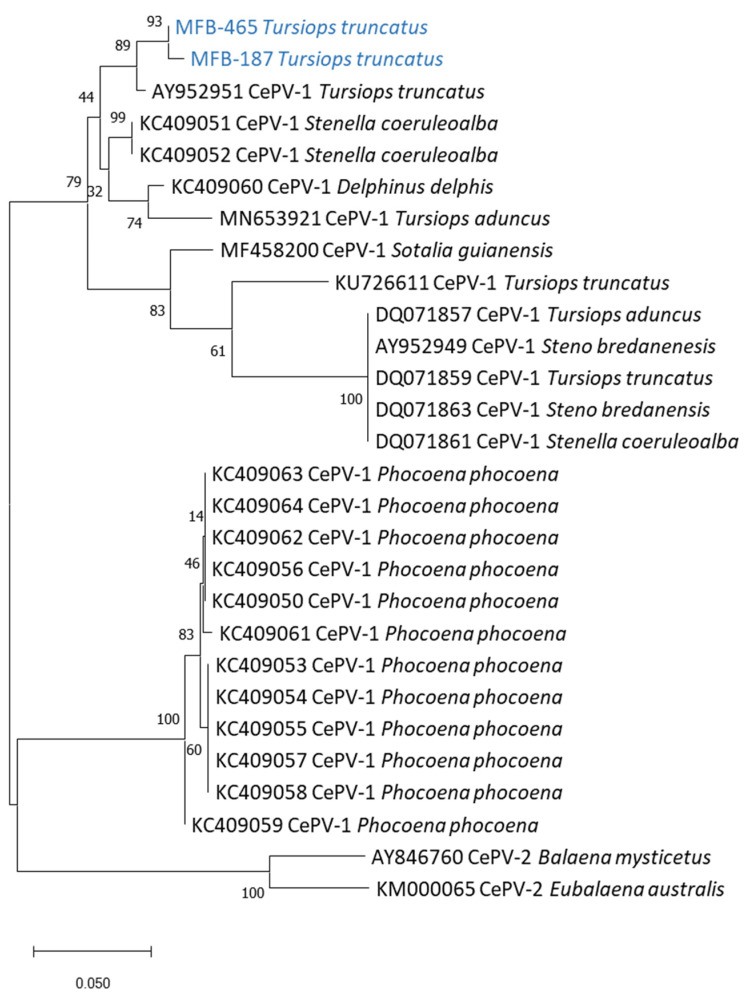
Phylogenetic analysis of CePV DNA topoisomerase nucleotide sequences obtained from tattoo skin lesions sampled in *T. truncatus* MFB-187 and MFB-465. The phylogenetic tree was created using maximum-likelihood methods based on the Tamura three-parameter model [27]. Numbers represent bootstrap values for each node, calculated with 5000 replicates. For sequences extracted from databases, sequence information corresponds to the virus GenBank accession number following the scientific name of the cetacean species in which the CePVs were detected. This analysis involved 28 nucleotide sequences. There was a total of 302 positions in the final dataset. Evolutionary analyses were conducted in MEGA X [28].

**Figure 3:**
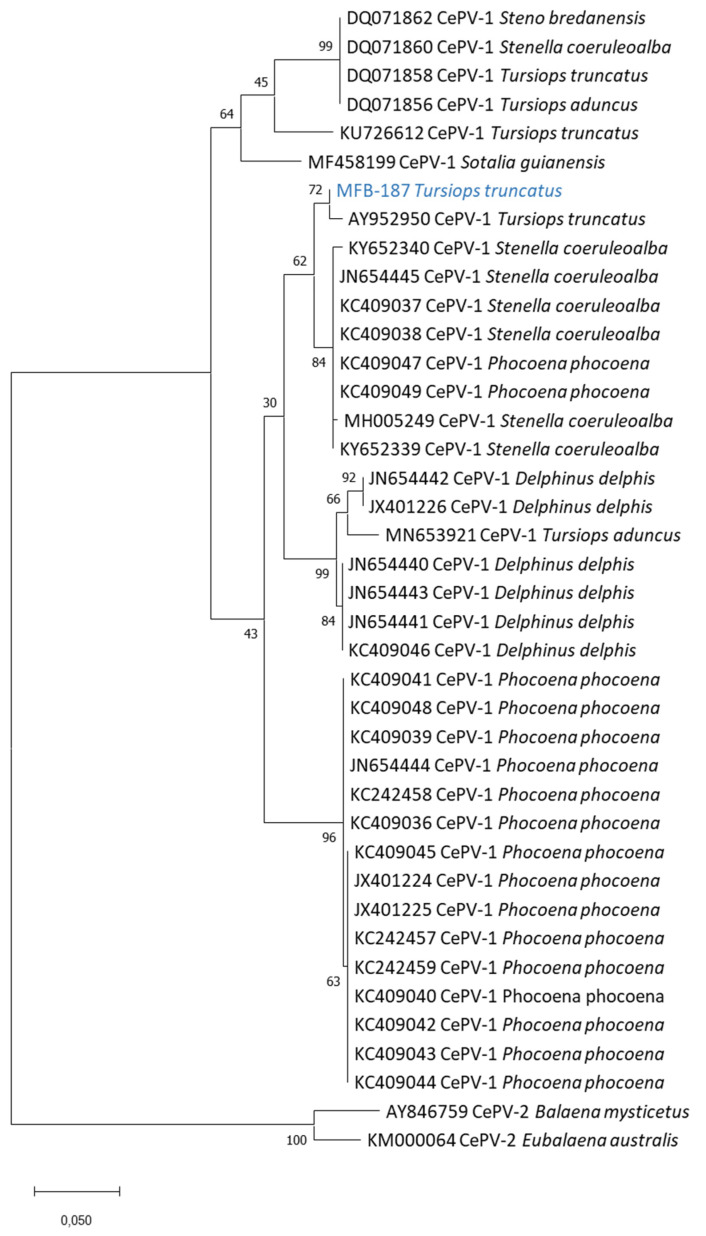
Phylogenetic analysis of CePV DNA polymerase nucleotide sequences obtained from tattoo skin lesions sampled in *T. truncatus* MFB-187. The phylogenetic tree was created using maximum-likelihood methods based on the Tamura three-parameter model [27]. Numbers represent bootstrap values for each node, calculated with 5000 replicates. For sequences extracted from databases, sequence information corresponds to the virus GenBank accession number following the scientific name of the cetacean species in which CePVs were detected. This analysis involved 40 nucleotide sequences. There was a total of 458 positions in the final dataset. Evolutionary analyses were conducted in MEGA X [28].

We tested 16 skin lesion samples collected in five Peruvian odontocete species in 1989–1995 (Table 1) with pan-poxvirus [19], pan-orthopoxvirus [22] and pan-parapoxvirus [23] PCR systems. None of the samples were positive using the pan-ortho or pan-parapoxvirus systems. Two were positive using the pan-poxvirus system: sample MFB-465, collected in an inshore female *Tursiops truncatus* (preserved in DMSO), and sample MFB-187, obtained from an offshore male *T. truncatus* (preserved in glutaraldehyde), both sexually immature and caught by fishermen off Cerro Azul (13°02′ S, 76°29′ W) in 1993. The sequencing of both amplicons from the pan-poxvirus system resulted in short and identical sequences: GATTACTTAGTTAGACGCGTTATAGATGAAAATAGAAGTGTTTTGTTATTTCATATTATGGG. Using the NCBI’s Nucleotide Blast tool (https://blast.ncbi.nlm.nih.gov/ (accessed on 1 August 2022)), we found that this sequence had only one match with sequence MN653921.1 Cetacean poxvirus 1 strain CePV-TA, i.e., the only complete CePV genome deposited in GenBank [9], sharing 61 identical nucleotides out of 62 (98.4% identity). The sequencing of the CePV DNA topoisomerase gene was successful for both samples, while the sequencing of the DNA polymerase gene was only possible for sample MFB-187. The phylogenetic trees including all CePV sequences available on GenBank ( Table 2) showed that both poxviruses from the offshore and inshore *T. truncatus* are closely related (99.3% estimates of evolutionary convergence between the sequences (EECS)), belong to the CePV-1 clade and are more closely related (98.4% EECS) to a poxvirus detected in a cutaneous lesion from a *T. truncatus* (AY952950 and AY952951) stranded in Florida [7] (Figure 2 and Figure 3). The nucleotide identity with the other Delphinidae CePVs, including those recovered from other *T. truncatus* populations, striped dolphins (*Stenella coeruleoalba*), short-beaked common dolphins (*Delphinus delphis*), Guiana dolphins (*Sotalia guianensis*) and rough-toothed dolphins (*Steno bredanensis*) [7,9,15,26], varied between 83.5% and 97.9% (Figure 2 and Figure 3). The phylogenetic trees also indicated that the poxviruses isolated in the two Peruvian *T. truncatus* were less related (81.1% EECS) to the harbor porpoise (*Phocoena phocoena*) CePVs and clearly differed from the CePV-2 clade, which comprises poxviruses of mysticetes (Figure 2 and Figure 3).

## 4. Discussion

This work has further confirmed the usefulness, specificity and performance of the recently developed pan-poxvirus system [19] for poxvirus discovery and rapid genus identification. Indeed, we succeeded in amplifying nucleic acids that had been preserved in DMSO and gluteraldehyde for about 30 years. CePV DNA could not be amplified in the tattoo skin lesion samples kept in formalin at room temperature, although poxviruses were observed by electron microscopy in some of them. This is likely the result of nucleic acid degradation induced by formalin [29]. The sequencing of the amplicons from the pan-poxvirus PCR system, although short in length, allowed the rapid detection of CePVs in tattoo skin lesions from two common bottlenose dolphins by-caught off Cerro Azul, Peru, in 1993, as expected. Thus, this pan-poxvirus PCR offers an alternative to the PCR tests developed by Bracht et al. in 2006 [7] that amplify regions of the DNA polymerase and DNA topoisomerase I genes and have been used and modified in various studies [8,13,15].

The Peruvian *T. truncatus* poxviruses belong to the CePV-1 clade, a heterogeneous clade that includes poxviruses isolated in Delphinidae and Phocoenidae and that is distantly genetically related to the CePV-2 clade that, until now, comprised only two poxviruses from a bowhead whale (*Balaena mysticetus*) and a southern right whale (*Eubalaena australis*) [7,14]. In the amplified region of the D6R (homologue *Monkeypox virus*) gene, their nucleotide sequence was identical and shared 98.4% identity with CePV-TA, the only CePV whose genome was completely sequenced [9]. In the phylogenetic trees obtained from the DNA polymerase and DNA topoisomerase I genes sequences, the Peruvian *T. truncatus* poxviruses were more closely related to a CePV-1 (AY952950 and AY952951) detected in a cutaneous lesion from a *T. truncatus* from Florida [7] (Figure 2 and Figure 3). They were less related to a CePV-1 strain (KU726612 and KU726612) recovered from a *T. truncatus* found dead in the Laguna Estuary, Brazil [15] (Table 2). The phylogenetic tree obtained from the DNA topoisomerase I gene sequences shows that the CePVs from the Peruvian *T. truncatus* were also very closely related to each other, although the dolphins belong to two different ecotypes (inshore and offshore) that are both morphologically and genetically distinct [16,28] and there is no evidence of the forming of mixed groups (K. Van Waerebeek, unpublished data). The testing of TSD lesions sampled in common bottlenose dolphins from other ocean provinces could provide more information into the phylogenetic relationships between their poxviruses. The Peruvian *T. truncatus* CePVs were more distantly related to harbor porpoise poxviruses, as described for other poxviruses of Delphinidae [8,9].

This is the first time that cetacean poxviruses are detected by DNA sequencing in free-ranging odontocetes from the Eastern Pacific. With the exception of CePVs from captive *T. aduncus*, all sequenced CePVs had been sampled in free-ranging dolphins and porpoises from the Atlantic Ocean, North Sea and Mediterranean Sea [7,8,9,13,15,26]. This further confirms the circulation of cetacean poxviruses in dolphins and Burmeister‘s porpoises from the Southeast Pacific, as already indicated by electron microscopy and serological data [11,17,18]. The prevalence of poxvirus skin disease (Figure 1a) was 41.6% in offshore *T. truncatus* caught off the Peruvian central coast in 1993–1994 [11]. Two inshore (including MFB-465) and six offshore *T. truncatus* from this region also had neutralizing serum antibodies against cowpoxvirus, sometimes with high titers [18]. These data suggest that there may be cross-immunity between different genera of chordopoxviruses. There have been no indications of any zoonotic danger posed by cetacean poxviruses, despite high prevalence levels in thousands of freshly dead dolphins and porpoises landed at Peruvian ports [21], which were handled unprotected by large numbers of fishers, fish market workers and researchers.

The poxviridae have some of the most complex and diverse genomic characteristics among viruses. Genomic studies tend to show that this family of viruses is probably as old and diverse as the animal kingdom. According to the Bayesian method of molecular clock dating [30], the genus *Avipoxvirus* is estimated to have been the first to differentiate among the chordopoxviruses 250,000 years ago. The very first poxvirus dates back to before the separation of vertebrates, as indicated by the presence of two different subfamilies in invertebrates (*Entomopoxvirinae*) and vertebrates (*Chordopoxvirinae*). The genomic structure is broadly similar between the different genera, but the gene content is host-dependent [31]. Sometimes, the same genus can show significant differences and mutations as a result of thousands of years of co-evolution, including mutations, deletions, insertions and horizontal transfers [21,32]. Our data further suggest that cetacean poxviruses have indeed co-involved with their hosts over a long time, with related viruses in Delphinidae from diverse ocean provinces (e.g., oceanic and coastal) and distant oceans as well as different clades in mysticetes and odontocetes and different lineages in the various odontocete families. They further support the establishment of a new genus for the poxviruses infecting cetaceans, as recommended by others [7,8,9,13].

## Figures and Tables

**Table 1:** Data on the cetaceans sampled for the tattoo skin lesions tested in this study, with the results of pan-poxvirus real-time PCR. Abbreviations: CT = cycle threshold, GA = glutaraldehyde, DMSO = dimethylsulfoxide, EM = electron microscopy, ND = not done.

Sample	Species		Year	Locality	Conservation	EM	Pan-Poxvirus
AGG-574	Long-beaked common dolphin	*Delphinus* cf. *capensis*	1991	Ancon	GA	ND	Neg
MFB-195	Long-beaked common dolphin	*Delphinus* cf. *capensis*	1993	Cerro Azul	Formalin	ND	Neg
MFB-297	Long-beaked common dolphin	*Delphinus* cf. *capensis*	1993	Cerro Azul	GA	ND	Neg
MFB-364	Short-finned pilot whale	*Globicephala macrorhynchus*	1993	Cerro Azul	DMSO	ND	Neg
MFB-001	Dusky dolphin	*Lagenorhynchus obscurus*	1989	Pucusana	Formalin	Poxvirus	Neg
KVW-1899	Dusky dolphin	*Lagenorhynchus obscurus*	1989	Pucusana	Formalin	ND	Neg
AGG-580	Dusky dolphin	*Lagenorhynchus obscurus*	1991	Ancon	GA	ND	Neg
AGG-663	Dusky dolphin	*Lagenorhynchus obscurus*	1992	Ancon	GA	ND	Neg
MFB-146	Dusky dolphin	*Lagenorhynchus obscurus*	1993	Cerro Azul	GA	ND	Neg
KVW-1864	Burmeister’s porpoise	*Phocoena spinipinnis*	1989	Pucusana	Formalin	ND	Neg
KVW-1863	Burmeister’s porpoise	*Phocoena spinipinnis*	1989	Pucusana	Formalin	ND	Neg
KVW-2283	Burmeister’s porpoise	*Phocoena spinipinnis*	1990	Pucusana	Formalin	Poxvirus	Neg
KOS-83	Burmeister’s porpoise	*Phocoena spinipinnis*	1993	Cerro Azul	Formalin	ND	Neg
KVW-2427	Burmeister’s porpoise	*Phocoena spinipinnis*	1995	San Jose	Formalin	ND	Neg
MFB-465	Common bottlenose dolphin	*Tursiops truncatus*	1993	Cerro Azul	DMSO	ND	21CT
MFB-187	Common bottlenose dolphin	*Tursiops truncatus*	1993	Cerro Azul	GA	Poxvirus	16CT

**Table 2:** Species, location, sample type, reference and GeneBank accession number (#) of the cetacean poxviruses used in the phylogenetic trees; NA: not available.

Species	Scientific Name	Location	Sample type	Dna Polymerase Genbank #	Dna Topoisomerase Genbank #	References	Clade
bowhead whale	B. mysticetus	USA (Alaska)	Killed	AY846759	AY846760	[7]	CePV-2
southern right whale	E. australis	Argentina	stranded	KM000064	KM000065	[14]	CePV-2
common dolphin	D. delphis	UK (Cornwall)	stranded	KC409046	KC409060	[8]	CePV-1
common dolphin	D. delphis	UK (Cornwall)	stranded	JN654440	NA	[13]	CePV-1
common dolphin	D. delphis	UK (Cornwall)	stranded	JN654441/42	NA	[13]	CePV-1
common dolphin	D. delphis	UK (Cornwall)	stranded	JN654443	NA	[13]	CePV-1
common dolphin	D. delphis	UK (Cornwall)	stranded	JX401226	NA	[13]	CePV-1
rough-toothed dolphin	S. bredanensis	USA (FL)	stranded	AY463004	AY952949	[7]	CePV-1
rough-toothed dolphin	S. bredanensis	USA (FL)	stranded	DQ071862	DQ071863	[7]	CePV-1
striped dolphin	S. coeruleoalba	UK (Dorset)	stranded	KC409037	KC409051	[8]	CePV-1
striped dolphin	S. coeruleoalba	USA (FL)	stranded	DQ071860	DQ071861	[7]	CePV-1
striped dolphin	S. coeruleoalba	UK (Cornwall)	stranded	JN654445	NA	[13]	CePV-1
striped dolphin	S. coeruleoalba	UK (Dorset)	stranded	KC409038	KC409052	[8]	CePV-1
striped dolphin	S. coeruleoalba	Italy (Toscana)	stranded	KY652339	NA	[26]	CePV-1
striped dolphin	S. coeruleoalba	Italy (Lazio)	stranded	KY652340	NA	[26]	CePV-1
striped dolphin	S. coeruleoalba	Spain (Mediterranean)	stranded	MH005249	NA	[15]	CePV-1
guiana dolphin	S. guianensis	Brazil (Rio de Janeiro)	stranded	MF458199	MF458200	[15]	CePV-1
indo-pacific bottlenose dolphin	T. aduncus	Hong Kong	captive	MN653921	MN653921	[9]	CePV-TA
indo-pacific bottlenose dolphin	T. aduncus	Hong Kong	captive	DQ071856	DQ071857	[7]	CePV-1
common bottlenose dolphin	T. truncatus	Brazil (Santa Catarina)	stranded	KU726612	KU726611	[15]	CePV-1
common bottlenose dolphin	T. truncatus	USA (FL)	stranded	AY952950	AY952951	[7]	CePV-1
common bottlenose dolphin	T. truncatus	USA (FL)	NA	DQ071858	DQ071859	[7]	CePV-1
harbor porpoise	P. phocoena	UK (Humberside)	stranded	KC409047	KC409064	[8]	CePV-1
harbor porpoise	P. phocoena	UK (Dorset)	stranded	KC409045	KC409059	[8]	CePV-1
harbor porpoise	P. phocoena	UK (West Sussex)	stranded	KC409043	KC409057	[8]	CePV-1
harbor porpoise	P. phocoena	UK (Devon)	stranded	KC409044	KC409058	[8]	CePV-1
harbor porpoise	P. phocoena	UK (Humberside)	stranded	KC409042	KC409056	[8]	CePV-1
harbor porpoise	P. phocoena	UK (Humberside)	stranded	KC409041	KC409055	[8]	CePV-1
harbor porpoise	P. phocoena	UK (Humberside)	stranded	KC409040	KC409054	[8]	CePV-1
harbor porpoise	P. phocoena	UK (Kent)	stranded	KC409039	KC409053	[8]	CePV-1
harbor porpoise	P. phocoena	UK (Northumberland)	stranded	KC409036	KC409050	[8]	CePV-1
harbor porpoise	P. phocoena	UK (Humberside)	stranded	Not used	KC409061	[8]	CePV-1
harbor porpoise	P. phocoena	UK (Humberside)	stranded	Not used	KC409062	[8]	CePV-1
harbor porpoise	P. phocoena	UK (Humberside)	stranded	KC409049	KC409063	[8]	CePV-1
harbor porpoise	P. phocoena	UK (Cornwall)	stranded	KC242457	NA	[13]	CePV-1
harbor porpoise	P. phocoena	UK (Cornwall)	stranded	KC242458	NA	[13]	CePV-1
harbor porpoise	P. phocoena	UK (Cornwall)	stranded	KC242459	NA	[13]	CePV-1
harbor porpoise	P. phocoena	UK (Cornwall)	stranded	JN654444	NA	[13]	CePV-1
harbor porpoise	P. phocoena	UK (Cornwall)	stranded	JX401224	NA	[13]	CePV-1
harbor porpoise	P. phocoena	UK (Cornwall)	stranded	JX401225	NA	[13]	CePV-1

## Data Availability

Samples were collected under Peruvian Ministry of Fisheries (*Ministerio de Pesquería*) Permit No. 064-93-PE/DNE′.
